# Feasibility and Acceptability Findings of an Energy Balance Data Repository of Children, Adolescents, and Young Adults with Cancer

**DOI:** 10.3390/jcm9092879

**Published:** 2020-09-06

**Authors:** Maria C. Swartz, Alaina K. Teague, Stephanie J. Wells, Theresa Honey, Min Fu, Kris M. Mahadeo, Laura S. Kabiri, Joya Chandra, Karen Moody, Keri Schadler

**Affiliations:** 1 Department of Pediatrics-Research, The University of Texas MD Anderson Cancer Center, 1515 Holcombe Blvd., Houston, TX 77030-4009, USA; mchang1@mdanderson.org (M.C.S.); akteague@UTMB.EDU (A.K.T.); SJWells1@mdanderson.org (S.J.W.); TAHoney@mdanderson.org (T.H.); jchandra@mdanderson.org (J.C.); 2 Department of Nutrition and Metabolism, The University of Texas Medical Branch, 301 University Blvd., Galveston, TX 77555-1124, USA; 3 Department of Pediatrics, The University of Texas MD Anderson Cancer Center, 1515 Holcombe Blvd., Houston, TX 77030-4009, USA; minfu@mdanderson.org (M.F.); KMMahadeo@mdanderson.org (K.M.M.); KMoody@mdanderson.org (K.M.); 4 Department of Kinesiology, Rice University, 6100 Main St. MS-545, Houston, TX 77005, USA; laura.kabiri@rice.edu

**Keywords:** neoplasms, fitness trackers, hematopoietic stem cell transplantation, nutritional status, child, exercise, feasibility studies, physical functional performance, adolescent, young adult

## Abstract

Cancer patients suffer changes in energy balance (EB), the combination of energy intake (nutrition) and energy expenditure (physical activity (PA)), which may influence cancer-related morbidity, mortality, and quality of life. Significant gaps remain in our understanding of the frequency and magnitude of these EB changes. Herein, we report on the feasibility and acceptability of a longitudinal repository of EB outcomes in children, adolescents and young adults (AYA) with cancer along the cancer continuum to fill these gaps. This EB repository includes PA, nutrition, and physical function (PF) parameters. PA data were gathered through activity trackers. Nutritional data were gathered through food diaries and micronutrient labs. PF was assessed with validated objective and patient-reported measures. Feasibility was achieved with >50% enrollment of eligible patients (*n* = 80, Mage = 18.1 ± 7.5); 26 were children with cancer and 54 were AYAs with cancer. An 88.75% retention rate indicated acceptability. Despite COVID-19 disruptions, >50% of participants provided completed data for PA and micronutrient labs as of April 2020. Food diaries and PF data collection experienced disruptions. Methodological adaptations are in progress to minimize future disruptions. Overall, our findings demonstrate that prospective EB assessments are feasible and acceptable among children and AYAs with cancer.

## 1. Introduction

Energy balance (EB), which collectively refers to the combination of energy intake (nutrition) and energy expenditure (physical activity (PA)), is an area of growing interest within the pediatric and adolescent and young adult (AYA) oncology communities [1,2,3]. This is largely due to evidence supporting the role of EB in patient outcomes [4]. Specifically, in children and AYA with cancer, malnutrition—including both over and undernutrition—is a risk factor for inferior cancer-related outcomes [4] and PA is associated with improved quality of life and physical function [5]. There are also emerging preclinical data that suggest that PA may improve tumor response to treatment [6,7] and that obesity may reduce tumor response to treatment via adipocyte signaling [8]. Both the intake and expenditure aspects of the EB equation as they relate to cancer outcomes will be reviewed briefly below.

Multiple factors, including cancer metabolic demand, cancer and treatment-related hormonal and metabolic disturbances, and complications of cancer and treatment that affect dietary intake and absorption, contribute to undernutrition [9]. Undernutrition is associated with inferior survival, reduced tolerance to chemotherapy, and a higher rate of infectious complications [10]. Dietary intake in children undergoing cancer treatment and in childhood cancer survivors has been shown to be similar to the general population with variable macronutrient intake, and poor consumption of fiber, vitamin D and calcium [11,12,13]. Observational studies have shown that children with leukemia that have low intakes of vitamin C, E and beta-carotene have increased risk for chemotherapy-related side effects [14]. Micronutrient laboratory analyses in children with various malignancies have revealed a high frequency of deficiencies in vitamins C, D and zinc [11]. In addition, malnutrition presenting as obesity is a risk factor for cancer-related late effects such as cardiovascular disease, metabolic syndrome, osteoporosis, and Type II diabetes mellitus [8]. To date, there is no universally accepted approach to nutritional support for children with cancer [15]. Screening and assessment for malnutrition is not standardized in young cancer patients. Decelerated physical growth (height and weight), obesity, micronutrient deficiencies and sarcopenia are not universally appreciated or addressed by pediatric oncology health care providers. Significant gaps exist in our understanding of the mechanisms of these malnourished states and there are no evidence-based guidelines for how to prevent or treat them [8]. To date, the development of dietary guidelines has been hampered by a lack of high-quality clinical trials that include nutritional variables, interventions, and endpoints in pediatric cancer patients and survivors. Thus, the emphasis of available studies is on general nutrition education for an overall healthy lifestyle. There remains a critical gap in understanding the precise nutritional needs of young cancer patients and survivors [10,16,17,18]. 

The story is similar regarding physical fitness. It is well documented that cancer patients and survivors are at risk for decreased physical fitness and decreased PA [19]. Young cancer patients and survivors experience physical and functional deconditioning at a faster rate when compared with siblings [20,21]. Cancer-related fatigue, cancer treatment-related side effects and organ toxicities, malnutrition, infectious morbidity, pain, and surgical interventions can all contribute to physical inactivity and poor physical function (PF) [22]. Furthermore, reduced muscle mass, or sarcopenia, can occur in both under and over nourished states of malnutrition in childhood cancer patients and is associated with reduced PF and premature aging in childhood cancer survivors [15]. The effects of poor physical fitness are particularly concerning due to the negative implications they have on patients’ and survivors’ ability to fulfill basic life roles (e.g., activities of daily living, personal care). These functional and activity limitations can further lead to other negative consequences, such as poor overall quality of life (QOL), poor mental health, unemployment, financial toxicity, and related comorbidities and mortality [23,24]. However, there is no accepted standardized approach to the screening or treatment of PA and PF declines experienced in young cancer patients and survivors throughout the care continuum [25].

Additionally, emerging evidence shows that physically active young cancer patients have better QOL [5,26,27]. PA in children with leukemia is associated with improved strength and flexibility, superior cardiopulmonary function, decreased fatigue and enhanced health-related QOL [5]. PA is also correlated with higher PF and mobility in childhood cancer survivors [27]. Given the increased risk in young cancer survivors for cardiovascular disease, diabetes mellitus type 2 and metabolic syndrome, exercise may emerge as a key factor in the mitigation of these late comorbidities. This has certainly been shown to be the case in adult cancer patients [26,28,29]. Furthermore, evidence from animal models suggests that moderate exercise may improve chemotherapy and checkpoint inhibitor efficacy, and prevent cardiac toxicity associated with anthracycline use [6,30,31,32]. While the data to support the efficacy of PA to improve health outcomes in childhood cancer patients are limited to a few, small, heterogeneous studies, evidence for safety and feasibility is strong. No deleterious effects of exercise interventions have been found in these children [26]. Regardless, there are no generally accepted exercise recommendations for pediatric cancer patients, and evidence for the optimal timing, modality, frequency and intensity of exercise is lacking [8].

A significant gap remains in our understanding of the factors that contribute to the EB-related morbidity observed in children with cancer and the effects of EB morbidity on important health-related outcomes. There is not yet comprehensive data on EB behaviors across the cancer continuum for most pediatric and AYA diagnoses. To begin to address this gap, we developed and implemented the Energy Balance Data Repository. The long term goals of implementing this data repository are to: (1) describe key elements of EB in this population, (2) facilitate the collection of EB variables and outcomes into other therapeutic clinical trials, (3) assess associations between EB, nutritional status, functional status, precondition status, cancer-related outcomes, and quality of life, and (4) generate hypotheses that will lead to developing and testing interventions to improve EB and subsequent health-related outcomes in this population. The purpose of this article is to report on the feasibility and acceptability of implementing this data repository in pediatric oncology patients and survivors at multiple time points along the cancer continuum. 

## 2. Experimental Section

The MD Anderson Energy Balance Data Repository was created to prospectively collect nutrition, physical activity and physical function measures on children and AYAs with cancer or who have survived cancer. Approval for this repository was obtained by the MD Anderson Institutional Review Board.

### 2.1. Feasibility and Acceptability Parameters

Feasibility and acceptability of the repository data collection methods are reported herein. Based on previous research regarding the components of feasibility studies [33,34,35], we defined feasibility as successful enrollment of ≥50% of the eligible participants approached [33] by clinical research coordinators at MD Anderson Cancer Center (MDACC). In addition, a qualitative measure of feasibility included a description of resources needed for data collection, including staffing, time, and logistical constraints. A retention rate of ≥80% of participants for at least 12 months defined the acceptability of the repository [33]. Acceptability was also defined as >50% of data collection completed from those consented to each repository component [34].

### 2.2. Informed Consent

All patients ≥ 18 years old were required to provide informed written consent, and children ≥ 7 years old were required to provide informed written assent for participation. Minor patients or patients without legal capacity must have had their legal guardian provide consent. Patients were re-consented after they have turned 18 years old. During the enrollment process, participants provided consent/assent for only the specific procedures they agreed to partake in. These options include: nutrition history, nutrient laboratory evaluation, PA, and PF. This design allowed for the collection of some of the data (e.g., only nutrition data or only PA data) or all data from each enrolled patient, for any period of time up to 3 years and was specifically intended to optimize participation rate by removing the need for all-or-nothing participation requirement. 

### 2.3. Inclusion and Exclusion Criteria

#### 2.3.1. Inclusion Criteria

Pediatric and AYA patients with a cancer diagnosis up to age 39 were eligible to participate. The American Cancer Society and the National Cancer Institute defined children with cancer as those diagnosed at age <15 years old. AYAs with cancer is defined as those diagnosed between the ages of 15 to 39 years old [2,3,36]. Patients are eligible for enrollment at any time from diagnosis through long-term survivorship, regardless of their participation in other studies. In fact, co-enrollment with other clinical trials is strongly encouraged, and, to date, 4 open clinical trials at MDACC require co-enrollment to the MDACC Energy Balance Data Repository in order to collect the energy balance outcomes targeted in the studies.

#### 2.3.2. Exclusion Criteria 

There were no specific exclusion criteria for overall participation as long as consent/assent is obtained.

### 2.4. Data Collection

**Figure 1:**
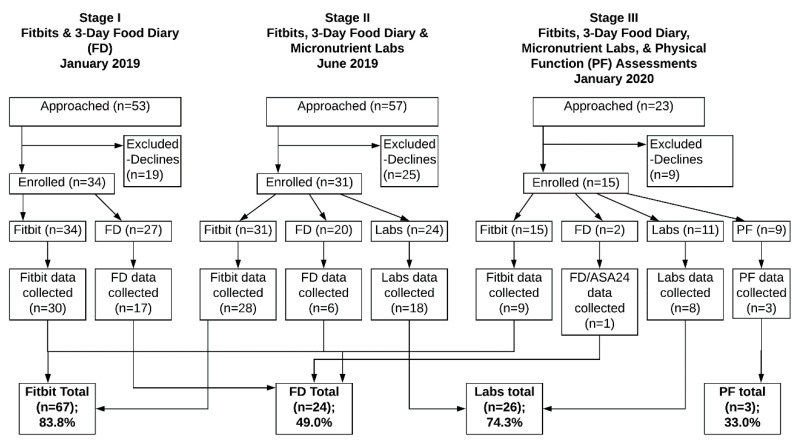
Flow diagram of the energy balance (EB) repository.

The repository was rolled out in 3 stages (Figure 1). The initial stage included the collection of only demographic and clinical data, PA via activity monitor (with optional written activity log) and nutrition history by self-reported food diaries. Recruitment started in January 2019. Stage two included the addition of a nutrient laboratory evaluation (June 2019), and the third stage included PF testing and the option of providing a nutrition history via self-report using a web-based tool instead of food diaries (January 2020). Activity monitor tracking was continuous, laboratory testing could occur up to 6 times per year, and all other data could be collected up to 4 times per year for up to 3 years. The last date of inclusion for data presented in the present study is 27 April 2020. 

#### 2.4.1. Clinical and Demographic Data

Demographic data were collected at baseline and included: age at enrollment, gender, race, and ethnicity. The clinical data collected at baseline through history and chart extraction included: diagnosis, presence or absence of relapse, presence or absence of metastases, dates and description of cancer treatment (surgery, chemotherapy/immunotherapy, radiation, stem cell transplant and other cellular therapies), preconditions, weight, height, BMI, performance score when available (i.e., Lansky–Karnofsky or Eastern Cooperative Oncology Group (ECOG) scores). Additional data extractions that are planned include vital status (e.g., death, last date of follow up).

#### 2.4.2. Diet and Nutrition Data

Diet history was obtained on consenting participants in stage I by self-report via 3-day food diaries, a validated and reliable tool for dietary research [37]. A registered dietitian or a trained research assistant provided participants and parents/caregivers with instructions on how to complete the food diaries at enrollment. These instructions included listing specific food items found in a recipe, portion sizes with amounts consumed, and the importance of recording a routine dietary intake, including a weekend day. Parents/caregivers could help patients complete the 3-day food record as needed. A registered dietitian could also assist patients/parents/caregivers in completing a 3-day food diary by phone if necessary. Food diaries could be completed as often as weekly, but no more than 4 times per year and could be returned electronically or at the next clinic visit. In stage III, participants were offered the option of using a self-administered 24-h (ASA24) dietary assessment tool in place of the 3-day food diaries. ASA24 is a validated web-based dietary assessment tool that provides automated assistance and uses the multi-pass method to obtain accurate 24-h dietary recall data [38,39,40,41]. The ASA 24 has also been shown to be comparable to interviewer led recalls in adolescents, implying that they can be used accurately in this population [42]. It was developed and is currently maintained by the Epidemiology and Genomics Program at the National Cancer Institute [43,44].

Micronutrient labs, added at stage II, could be drawn up to weekly and no more than 6 times in one year. Blood for the study was preferably drawn prior to a chemotherapy cycle during a routine blood draw and ideally within 24 h following a diet history. No extra venipunctures or central venous catheter (CVC) accesses were permitted. The volume of blood needed to run the micronutrient analyses was approximately 17.5 mL, and total blood drawn could not exceed 3 mL/kg/day in compliance with the MDACC policy for phlebotomy in children. A total of ten micronutrients were assessed. These included vitamins A, B6 (pyridoxine), B12, C, D (25-hydroxy), ceruloplasmin, serum copper, zinc (plasma), folate, and beta-carotene. The PI or collaborators placed the orders to run the labs in the MDACC EPIC system as part of standard care laboratory evaluation.

#### 2.4.3. Physical Activity Data

PA was tracked with a Fitbit (e.g., Alta HR or Inspire HR) for adults and children who had wrists large enough for the device wristband. For children with wrists too small for the Alta HR or Inspire HR, Fitbit Ace 2 devices (designed for children 6 years old and above) were used. Fitbits are wrist-worn, watch-like devices that utilize an accelerometer and heart rate monitor to determine PA duration and intensity on a continuous basis during wear. Fitbit devices were chosen because of the existing expertise within MDACC, because of the availability of a health insurance portability and accountability act of 1996 (HIPAA) compliant cloud-based data collection platform (Fitabase; www.fitabase.com), and because of the ease of use for patients.

Fitbits and chargers were given to participants at no cost, and the participants were required to enroll their device via the Fitbit website and consent to allowing the collection of activity data via Fitabase. Participants used identification numbers, not names, to enroll their device. Participants were asked to sync data to a smart phone or web-enabled device, via either Fitbit.com or the Fitbit app. Study personnel had access to data via Fitabase.

To address non-adherence with uploading data, communication was done via the email address and/or mobile number that the parent or patient provided at the time of enrollment. An email, text, or phone call (depending on preference) was used to encourage syncing the device. Study staff monitored data uploads and, if large gaps (~2 weeks) in data collection were noted, the participant was contacted to address barriers to syncing data and to promote increased adherence to data collection.

For patients without access to the internet, and with parent/guardian permission, study staff were allowed to provide instructions regarding the device and the website interface to the patient’s school personnel so that the patient could participate through school internet access. Alternatively, a patient could participate in Fitbit data collection through use of tablet devices owned by and stored within the Division of Pediatrics at MDACC. To date, no patient on the study has needed these options, as all had internet access. In addition to Fitbit PA tracking, the study allowed for the use of Actigraph accelerometers or written PA logs at the discretion of the co-enrollment PI. To date, only Fitbit PA data collection has been used.

#### 2.4.4. Physical Function Data 

We adopted the World Health Organization’s definition of the PF construct. The international classification of function, disability, and health (ICF) model describes the need to assess function in three categories: at the body level (impairment of body structure and function), at the individual level (activity limitations), and the societal level (participation restrictions, quality of life) [45,46]. Table 1 provides a summary of the physical function domains and the associated assessments used in this protocol. These assessments were chosen because they are validated and recommended by the Academy of Pediatric Physical Therapy for monitoring functional development and functional changes based on the ICF model [47,48]. Assessment was requested no more than 4 times per year.

#### 2.4.5. Off Study Criteria

Participants were taken off study due to death, or if they were lost to follow-up (no scheduled appointments and no response to follow-up calls monthly × 3 months), or withdrawal of consent for any further data submission.

### 2.5. Statistical Analysis 

Descriptive statistics were used to analyze quantitative feasibility and acceptability data. Qualitative feasibility information about the resources, time, and logistics of data collection was gathered through staff verbal and written reports.

## 3. Results

Of the 133 eligible patients approached, a total of 80 participants (60%) consented to at least one part of the EB data repository, demonstrating that longitudinal EB data collection in children and AYAs with cancer is feasible. The enrollment and data collection progress is summarized in Figure 1.

Descriptive characteristics of study participants are depicted in Table 2. Thus far, 26 patients are considered as pediatric cancer patients (diagnosed < 15 years old) and 54 patients are AYAs with cancer (diagnosed between ages 15 to 39). The mean adult BMI for those ≥ 20 years old indicated that these cancer patients and survivors were overweight [49]. Of cancer patients and survivors < 20 years old, 35.4% were obese with BMI ≥ 95th percentile [49]. The majority of participants did not have relapse or metastasis. The performance status using either the Lansky–Karnofsky or the ECOG scores indicated that the majority were either fully active/asymptomatic or had minor restriction/symptomatic but ambulatory. However, performance status was only reported in the electronic medical record among 41 out of 80 enrolled participants. 

Of the 80 participants who consented, 32 were co-enrolled on another study that incorporated the data from this repository into its outcomes. In total, 1.5 full-time clinical research coordinators (CRCs) were needed to enroll and collect data on 80 patients over 16 months.

The retention rate of consented participants through April 2020 was 88.75%, indicating that this form of EB data collection is acceptable in this patient population. Nine participants dropped out of the study. Two participants died from cancer progression and one from pneumonia over the course of the study, five were taken out due to parent/guardian request, and one was taken out due to PI decision after a report from the participant’s family indicating lack of time to participate in the study. The number of participants consented to each component of the data repository and the number who completed data collection are listed in Table 3 and described below.

Nutrition data collection was feasible in terms of enrollment but less acceptable than other components of the study. Across stages I, II, and III, 49 of 80 participants (61%) consented to food diaries, and, of those, 49% completed the data collection. Dietitian effort (5% of full-time effort) was needed to train clinical research coordinators on the paper-based food diary methods. ASA24 nutrition diaries were added as an option in stage III and to date, one patient has consented to and completed ASA24 food recall. After micronutrient laboratory analysis was added in stage II, 76.1% of patients agreed to micronutrient laboratory analysis, and, of those, 74.3% completed at least 1 timepoint. There was difficulty scheduling some micronutrient laboratory blood draws in cases where patients did not have future clinical appointments scheduled or when there was a sudden change in a patient’s lab appointment date due to alterations in his/her chemotherapy schedule or clinical status. On occasion, micronutrient levels were unable to be measured due to hemolysis. 

**Figure 2:**
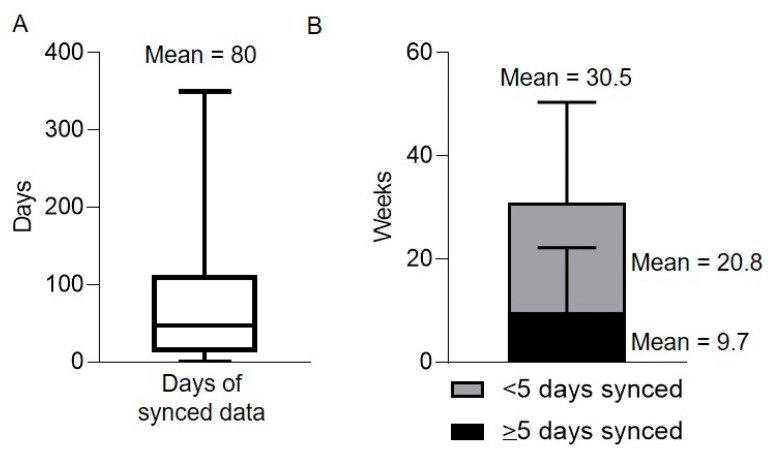
Fitbit data quantity and quality varied between patients. (**A**) Total number of days of Fitbit data synced (*n* = 80 patients); (**B**) Total number of weeks on study separated by the number of weeks with ≥5 days of synced data (black) and the number of weeks with <5 days of synced data (gray). Bars show the mean ± standard deviation, *n* = 80 patients.

Enrollment to the PA data collection was the most feasible of all components of the study. Across three stages, 100% of the recruited participants consented to Fitbit data collection. A total of 75 participants (93.8%) connected their Fitbit to Fitabase and 83.8% of those who connected the device synced it to the database to allow for data collection. Of the 67 patients who synced their data, the mean number of days of synced data was 80, with a range of 0 to 350 (Figure 2a). This equaled a mean of 30.5 weeks on study (Figure 2b). While patients were on study for an average of 30.5 weeks, the mean number of weeks before the final sync was 20.8, indicating that patients stopped syncing data prior to being removed from the study. Of the 30.5 weeks that the average participant was on study, the mean number of weeks with at least 5 days of synced data was 9.7, indicating that patients frequently did not wear and sync the Fitbit for several days per week (Figure 2b). If patients lost their Fitbit within the first six weeks of data collection, the Fitbit was replaced. If the Fitbit was lost after the first 6 weeks, the Fitbit was not replaced and PA data collection stopped. In some cases, the Fitbit was not lost but a patient chose to stop wearing and/or to stop syncing the Fitbit due to becoming significantly more ill or due to changing circumstances as the patient moved beyond the active treatment phase. 

Sixty percent of enrolled participants in stage III consented to the functional assessments, and 33% of those were able to complete the assessments. One inpatient participant declined to participate after consent due to lack of energy and anxiety about their condition, another was experiencing severe pain that prevented participation, one assessment was postponed due to the COVID-19 outbreak, and another decided to receive treatment at a different medical facility. We did not have the same difficulty among the outpatient participants. We found that it was easier to schedule the functional assessments around their upcoming clinical visits. However, there was one outpatient who did not respond to calls or emails from study staff. The aforementioned challenges in data collection are summarized in Table 4.

## 4. Discussion

The aim of this report was to describe the feasibility and acceptability of implementing a repository of EB data in young cancer patients and survivors. The greater than 50% recruitment of eligible participants and the staff effort required indicated feasibility of this repository. In addition, the >80% retention rate and completion of data components (e.g., PA and micronutrient labs) by >50% of consented participants indicated the overall acceptability of the study. It is important to note that, due to COVID-19, an institution-wide complete restriction of all in-person contacts for non-therapeutic studies was implemented, which hindered dietary data collection on one inpatient as well as physical function assessments on three outpatients. In addition, this restriction also negatively impacted recruitment rate and overall data collection due to the reassignment of staff, restricted campus access, and restrictions of in-person patient contacts for non-therapeutic research purposes. Both the paper 3-day food diaries and the PF assessments were completed by <50% of patients who consented, indicating significant barriers at the present time, which may be in part due to these COVID-19 social distancing measures. It is expected that once COVID19 restrictions are completely lifted, adherence with these aspects will improve greatly. 

Our results are comparable to previous studies that have used similar methods of EB data collection, including the use of wearable fitness trackers in young adult cancer patients, and found them to be feasible and acceptable [50]. However, the previous study did not include a nutrition component. To our knowledge, no report of the use of Fitbit wearable activity trackers and nutrition (energy balance parameters) to prospectively follow children and AYAs with cancer currently exists and ours is the first to report on the feasibility of an EB repository. We have added to the literature by developing methods to efficiently create an EB data repository with standard clinical data points that allow for a large data set to be created and to continue accruing data that would be used to develop and test a hypothesis. To improve the data collection utilizing Fitbits, the rate of Fitbit device connection to Fitabase could be improved by ensuring that all devices are connected during the same appointment as when the device is given to the patient. One limitation of Fitbit data collection is the effort needed by study personnel to remind patients to charge the device and sync regularly. While somewhat unavoidable, the rate of device charging and syncing could be improved with more support from non-study personnel, such as nurses and physicians, to encourage patient participation, coupling it with automated email and text reminders. 

Within the nutrition component of this study, micronutrient lab draws had the highest acceptability among the participants. It also required the least staffing time. Paper-based data collection of nutrition information was labor-intensive and required additional staff time. In addition, dietary information of inpatients is best collected in real time by on-site study personnel. In order to ensure the accuracy of the dietary data using the 3-day food diaries, the best practice is for a dietitian to review the food record for missing information (i.e., serving size, food description, preparation method, and mixed food ingredients) within one week of the receipt of the food diaries [37]. Therefore, we added the use of ASA24, an automated multi-pass method, to collect 3-day food diaries in stage III to reduce the time needed for a dietitian to complete an extensive review. Additionally, recent reviews have shown that the multi-pass method is the most valid method of assessing energy intake in children aged 4–11 years when compared against the doubly labeled water method of assessing total energy expenditure, known as the gold standard [40,41]. 

At present, we do not have enough data to compare the acceptance of ASA24 to 3-day food diaries, as only one patient to date consented to and completed an ASA24 log. The ASA24 has been shown to be feasible and acceptable to adolescents in other studies [42]. However, using the ASA24 data collection method also has limitations, including the requirement for internet access. One patient encountered challenges in submitting the ASA24 log using a mobile phone with ASA24 version 2018, which is supposed to be compatible with mobile devices. Additionally, the alignment between micronutrient lab draws, collection of food diaries (3-day food diaries or ASA24) and extraction of nutritional information for those on nutrition support require personnel time. These limitations indicate a need for alternative methods to be used simultaneously within EB repository studies to obtain accurate nutrition data within the child and AYA cancer patient population. 

Although PF assessment is highly accepted by patients, scheduling the assessments around diagnostic testing or chemotherapy session is difficult, particularly in the inpatient setting when patients may be too ill to participate. PF data collection timing needs to be more flexible to allow for inpatient participation given the waxing and waning of patients’ clinical status and the frequency of other tests and interventions needed in the inpatient setting. Lastly, due to COVID-19-related restrictions on in-person contact, we could not schedule PF assessments with three additional patients (of the nine enrolled). It is expected that once COVID19 restrictions are completely lifted, PF adherence will improve greatly. 

The logistical challenges experienced during the data collection from the first 80 patients of this study have presented some barriers to data collection that can be improved upon moving forward. One example of this would be automated text and email reminders that could cue patients to sync Fitbits. The addition of virtual physical function assessment may also improve the feasibility of data collection for this component. Other data collection, such as the ASA24, will likely be improved by loaning tablets to inpatients who are on the study during the time in which they are meant to complete the ASA24.

Our findings for the present study need to be considered in light of some limitations. First, the feasibility of using Fitbit is a resource that our hospital has committed to providing and funding. Therefore, obtaining Fitbits and subscribing to the Fitabase tracking system would likely be a challenge elsewhere. Second, it is not clear how generalizable this broad eligibility design will be at other institutions because significant time (and thus cost) will be required before a sufficient number of patients with one particular diagnosis to draw conclusions are enrolled. Third, within our study, changing from 3-day food recalls to the ASA24 system presents challenges to combining nutrition data from the original data collection mechanism and the new one (ASA24). Lastly, we did not gather information about the types of PAs patients participated in prior to and are currently participating in during our data collection, which would help inform our findings in sports and other PAs that are likely relevant to our findings regarding PA and PF. This is a limitation we have shared with other investigators who are co-enrolling patients on the EB repository study. We encourage the collection of PA history for their studies.

Despite the challenges, a strength of this repository study design is the flexibility of data collection, which is intended to promote the use of co-enrollment on this study with other ongoing studies. By co-enrollment on this EB data repository study, EB variables and endpoints can be easily incorporated into cancer therapeutic and supportive care trials. As such, we have used the broadest possible eligibility criteria (previously published studies focus on one diagnosis or a group of similar diagnoses, such as bone tumors or leukemias) and have created a repository of data, which, to our knowledge, does not exist elsewhere. 

## 5. Conclusions

Our data suggest that longitudinal EB assessments are feasible and acceptable in young cancer patients and survivors, particularly with the use of wearable activity trackers, 3-day food diaries, laboratory evaluations, and a flexible enrollment strategy. In the future, we plan to implement the refined EB data repository protocol in other institutions to allow for a broader data set. This data can facilitate an understanding of associations between EB variables and other therapeutic trial outcomes in children and AYAs with cancer. This will lead to hypotheses for EB interventions to be used in future pediatric/AYA cancer trials. The long-term goal is to develop an evidence base for EB interventions to improve outcomes in the pediatric and AYA cancer population.

## Figures and Tables

**Table 1:** Summary of domains being measured and the associated assessments [46,47].

Domains	Assessments		Purpose
The body level measures			
Body size	Skinfold measurement, circumference (waist, arm, and calf area)		Evaluate muscle mass development
Flexibility	Sit and Reach		Evaluate the flexibility of the lower back and hamstring muscles
The individual level measures			
Motor skills	Tests for <18 years old	Tests for ≥18 years old	Evaluate movement function
	Bruininks-Oseretsky Test of Motor Proficiency (BOT-2) Functional Gait Assessment (FGA)	FGA and Berg Balance Scale	
Performance level	Tests for <18 years old	Tests for ≥18 years	Evaluate strength, general mobility and function, and neuromuscular coordination
	Hand grip dynamometry TUG-3m 30 s chair stand BOT-2 Standing Long Jump (SLJ)	Hand grip TUG-3m 30 s chair stand SLJ	
Cardiovascular Fitness	6-min walk test (6-MWT) Borg Scale of Perceived Exertion (part of the 6-MWT) 20-m shuttle run (VO2 max)		Evaluate aerobic fitness and stamina
The societal level measures			
Self-reported QOL	Tests for <18 years old	Tests for ≥18 years old	Evaluate patient and survivors’ QOL
	PedsQL Cancer Module Wee-Functional Independence Measure (FIM)	PedsQL Cancer Module FIM	

**Table 2:** Summary of study participant characteristics (*n* = 80).

Participant Characteristics	Mean ± SD	
Age at enrollment	18.1 ± 7.5 Range: 4–36	
BMI (kg/m^2^) for those ≥20 years old (*n* = 30)	26.6 ± 11.1	
	*n* (%) aged <15 years	*n* (%) aged 15–39 years
Children with cancer	26 (33%)	N/A ^†^
AYAs with cancer	N/A ^†^	54 (67%)
Gender		
Male	17 (65.4%)	26 (48.1%)
Female	9 (34.6%)	28 (51.9%)
Race/Ethnicity		
Non-Hispanic White	11 (42.3%)	22 (40.7%)
African American	4 (15.4%)	3 (5.6%)
Hispanic	6 (23.1%)	23 (42.6%)
Asian	2 (7.7%)	4 (7.4%)
Other	2 (7.7%)	2 (3/7)
Not reported	1 (3.8%)	0
BMI percentile ^‡^		
≥95th percentile	5 (19.2%)	12 (22.2%)
≥85th to <95th percentile	2 (7.7%)	2 (3.7%)
5th to <85th percentile	17 (65.4%)	7 (17%)
<5th percentile	2 (7.7%)	1 (1.9%)
Not applicable	0	32 (59.3%)
Top 6 cancer diagnoses		
Acute lymphoblastic leukemia (ALL)	9 (34.6%)	12 (22.2%)
Acute myeloid leukemia (AML)	2 (7.7%)	7 (13%)
Wilms tumor	2 (7.7%)	0
Sarcoma	7 (26.9)	15 (27.8)
Osteosarcoma	5 (19.2%)	4 (7.4%)
CNS Tumor	0	4 (7.4%)
Other	1 (3.8%)	12 (22.2%)
Metastasis status		
No	20 (76.9%)	38 (70.4%)
Yes	5 (19.2%)	12 (22.2%)
Not applicable	1 (3.8%)	4 (7.4%)
Relapse status		
No	20 (76.9%)	37 (68.5%)
Yes	5 (19.2%)	13 (24.1%)
Not applicable	1 (3.8%)	4 (7.4%)
Lansky–Karnofsky score (*n* = 29)		
60	0	1 (1.9%)
70	0	2 (3.7%)
80	1 (3.8%)	3 (5.6%)
90	4 (15.4%)	12 (22.2%)
100—Fully active	4 (15.4%)	2 (3.7%)
Not reported	17 (65.4%)	34 (63%)
ECOG score (*n* = 12)		
Asymptomatic	0	5 (9.3%)
Symptomatic, but completely ambulatory	1 (3.8%)	6 (11.1%)
Not reported	25 (96.2%)	43 (79.6%)
Co-enrollment		
Therapeutic trials	0	0
Non-therapeutic trials	10 (38.5%)	22 (40.7%)
No co-enrollment	16 (61.5%)	32 (59.3%)

^†^ N/A: Not applicable; ^‡^ Based on the BMI-for-age percentile on a Centers for Disease Control and Prevention (CDC) BMI-for-age growth chart for children and teens aged 2 to 19 years old;

**Table 3:** Acceptability: participation rate and the completion of data collection.

Repository Component	Total Number of Participants Consented	Participation Rate: % of Recruited Who Consented	Compliance Rate: Percent of Consented Who Completed Data Collection
Physical Activity	Patient logs: *n* = 42 Fitbit: *n* = 80	Patient Logs = 52.5% Fitbit = 100%	% of Fitbits connected to Fitabase = 93.8% (75 connected) % of Fitbits collecting data = 83.8% (67 connected and collected data) Patient logs = N/A *
Nutrition	Food diaries: *n* = 49 Labs: *n* = 35 (out of 46 recruited after labs added to the study) ASA24 = 1 consented (out of 15 recruited after ASA24 was added to the study)	Food Diaries = 61.3% Labs = 76.1%	Food Diaries = 49% (23 paper recalls and 1 ASA 24 completed) Labs = 74.3% (26 completed labs)
Physical Function	Physical Function Tests: *n* = 9 (out of 15 recruited after physical function added to study)	Physical Function: 60%	Physical function tests = 33% (3 tests completed)

* Patient logs are an alternative to Fitbits to be used per PI decision at this point, and time no logs have been used by any participants.

**Table 4:** Feasibility: resources and logistics including time usage for 1.5 full-time Clinical Research Coordinators (CRCs).

Repository Component	Data Collection Challenges and Staff Time Descriptions
Recruitment	One and a half full-time equivalence Clinical Research Coordinators (CRCs) were required to review clinic schedules and recruit in clinic and remotely; CRCs spend 60% time on recruitment activities.
Physical Activity	Missing data in Fitabase/Fitbits not synced to database. CRCs spend 30% time texting, emailing, and following up with participants encouraging them to charge the Fitbit and to sync the data.
Nutrition	Inability to accurately do a 3-day food record for patient on intravenous nutrition. ASA24 challenges with mobile phone device. Lag time for delivery of paper diet recalls to dietitian for review. Difficulty scheduling patients for lab draws. Missing micronutrient levels for one participant. (Possible reasons include: missed draws, hemolysis, or substance interference.) CRCs spend 20% time on scheduling labs and review labs Research dietitians spend 5% time to review paper-based food diaries and resolve ASA24 issues.
Physical Function	Inpatient participants difficult to schedule due to acute illness. Contact restrictions due to COVID-19. Team members spend 10% time scheduling and conducting functional assessments.
Data entry	CRCs spend 30% time doing data entry

## References

[1] Hall K.D., Heymsfield S.B., Kemnitz J.W., Klein S., Schoeller D.A., Speakman J.R. (2012). Energy balance and its components: Implications for body weight regulation. Am. J. Clin. Nutr..

[2] Tam C.S., Ravussin E. (2012). Energy balance: An overview with emphasis on children. Pediatr. Blood Cancer.

[3] American Cancer Society (2020). Cancer Facts & Figures 2020.

[4] Schadler K.L., Kleinerman E.S., Chandra J. (2017). Diet and exercise interventions for pediatric cancer patients during therapy: Tipping the scales for better outcomes. Pediatr. Res..

[5] Baumann F.T., Bloch W., Beulertz J. (2013). Clinical exercise interventions in pediatric oncology: A systematic review. Pediatr. Res..

[6] Schadler K.L., Thomas N.J., Galie P.A., Bhang D.H., Roby K.C., Addai P., Till J.E., Sturgeon K., Zaslavsky A., Chen C.S. (2016). Tumor vessel normalization after aerobic exercise enhances chemotherapeutic efficacy. Oncotarget.

[7] Florez Bedoya C.A., Cardoso A.C.F., Parker N., Ngo-Huang A., Petzel M.Q., Kim M.P., Fogelman D., Romero S.G., Wang H., Park M. (2019). Exercise during preoperative therapy increases tumor vascularity in pancreatic tumor patients. Sci. Rep..

[8] Joffe L., Ladas E.J. (2020). Nutrition during childhood cancer treatment: Current understanding and a path for future research. Lancet Child Adolesc. Health.

[9] Gaynor E.P., Sullivan P.B. (2015). Nutritional status and nutritional management in children with cancer. Arch. Dis. Child.

[10] Selwood K., Ward E., Gibson F. (2010). Assessment and management of nutritional challenges in children’s cancer care: A survey of current practice in the United Kingdom. Eur. J. Oncol. Nurs..

[11] Morrell M.B.G., Baker R., Johnson A., Santizo R., Liu D., Moody K. (2019). Dietary intake and micronutrient deficiency in children with cancer. Pediatr. Blood Cancer.

[12] Ladas E.J., Orjuela M., Stevenson K., Cole P.D., Lin M., Athale U.H., Clavell L.A., Leclerc J.M., Michon B., Schorin M.A. (2016). Dietary intake and childhood leukemia: The Diet and Acute Lymphoblastic Leukemia Treatment (DALLT) cohort study. Nutrition.

[13] Zhang F.F., Saltzman E., Kelly M.J., Liu S., Must A., Parsons S.K., Roberts S.B. (2015). Comparison of childhood cancer survivors’ nutritional intake with US dietary guidelines. Pediatr. Blood Cancer.

[14] Kennedy D.D., Tucker K.L., Ladas E.D., Rheingold S.R., Blumberg J., Kelly K.M. (2004). Low antioxidant vitamin intakes are associated with increases in adverse effects of chemotherapy in children with acute lymphoblastic leukemia. Am. J. Clin. Nutr..

[15] Barr R.D., Ladas E.J. (2020). The role of nutrition in pediatric oncology. Expert Rev. Anticancer Ther..

[16] Wu Y.P., Yi J., McClellan J., Kim J., Tian T., Grahmann B., Kirchhoff A.C., Holton A., Wright J. (2015). Barriers and Facilitators of Healthy Diet and Exercise Among Adolescent and Young Adult Cancer Survivors: Implications for Behavioral Interventions. J. Adolesc. Young Adult Oncol..

[17] Moyer-Mileur L.J., Ransdell L., Bruggers C.S. (2009). Fitness of children with standard-risk acute lymphoblastic leukemia during maintenance therapy: Response to a home-based exercise and nutrition program. J. Pediatr. Hematol. Oncol..

[18] Ward E.J., Henry L.M., Friend A.J., Wilkins S., Phillips R.S. (2015). Nutritional support in children and young people with cancer undergoing chemotherapy. Cochrane Database Syst. Rev..

[19] Antwi G.O., Jayawardene W., Lohrmann D.K., Mueller E.L. (2019). Physical activity and fitness among pediatric cancer survivors: A meta-analysis of observational studies. Support. Care Cancer Off. J. Multinat. Assoc. Support. Care Cancer.

[20] Arora M., Sun C.L., Ness K.K., Teh J.B., Wu J., Francisco L., Armenian S.H., Schad A., Namdar G., Bosworth A. (2016). Physiologic Frailty in Nonelderly Hematopoietic Cell Transplantation Patients: Results From the Bone Marrow Transplant Survivor Study. JAMA Oncol..

[21] Armenian S.H., Gibson C.J., Rockne R.C., Ness K.K. (2019). Premature Aging in Young Cancer Survivors. J. Natl. Cancer Inst..

[22] Braam K.I., van der Torre P., Takken T., Veening M.A., van Dulmen-den Broeder E., Kaspers G.J. (2016). Physical exercise training interventions for children and young adults during and after treatment for childhood cancer. Cochrane Database Syst. Rev..

[23] Ketterl T.G., Syrjala K.L., Casillas J., Jacobs L.A., Palmer S.C., McCabe M.S., Ganz P.A., Overholser L., Partridge A., Rajotte E.J. (2019). Lasting effects of cancer and its treatment on employment and finances in adolescent and young adult cancer survivors. Cancer.

[24] Wilson C.L., Howell C.R., Partin R.E., Lu L., Kaste S.C., Mulrooney D.A., Pui C.H., Lanctot J.Q., Srivastava D.K., Robison L.L. (2018). Influence of fitness on health status among survivors of acute lymphoblastic leukemia. Pediatr. Blood Cancer.

[25] Stubblefield M.D., Schmitz K.H., Ness K.K. (2013). Physical Functioning and Rehabilitation for the Cancer Survivor. Semin. Oncol..

[26] Huang T.-T., Ness K.K. (2011). Exercise Interventions in Children with Cancer: A Review. Int. J. Pediatr..

[27] Rustler V., Hagerty M., Daeggelmann J., Marjerrison S., Bloch W., Baumann F.T. (2017). Exercise interventions for patients with pediatric cancer during inpatient acute care: A systematic review of literature. Pediatr. Blood Cancer.

[28] Mishra S.I., Scherer R.W., Snyder C., Geigle P., Gotay C. (2014). Are exercise programs effective for improving health-related quality of life among cancer survivors? A systematic review and meta-analysis. Oncol. Nurs. Forum.

[29] Kirkham A.A., Davis M.K. (2015). Exercise Prevention of Cardiovascular Disease in Breast Cancer Survivors. J. Oncol..

[30] Lemke D., Pledl H.-W., Zorn M., Jugold M., Green E., Blaes J., Löw S., Hertenstein A., Ott M., Sahm F. (2016). Slowing down glioblastoma progression in mice by running or the anti-malarial drug dihydroartemisinin? Induction of oxidative stress in murine glioblastoma therapy. Oncotarget.

[31] Betof A.S., Lascola C.D., Weitzel D.H., Landon C.D., Scarbrough P.M., Devi G.R., Palmer G.M., Jones L.W., Dewhirst M.W. (2015). Modulation of Murine Breast Tumor Vascularity, Hypoxia, and Chemotherapeutic Response by Exercise. J. Natl. Cancer Inst..

[32] Zheng X.I., Cui X.-X., Gao Z.H.I., Zhao Y., Shi Y.I., Huang M.-T., Liu Y.U.E., Wagner G.C., Lin Y., Shih W.J. (2011). Inhibitory effect of dietary atorvastatin and celecoxib together with voluntary running wheel exercise on the progression of androgen-dependent LNCaP prostate tumors to androgen independence. Exp. Ther. Med..

[33] Hawkins J., Charles J.M., Edwards M., Hallingberg B., McConnon L., Edwards R.T., Jago R., Kelson M., Morgan K., Murphy S. (2019). Acceptability and Feasibility of Implementing Accelorometry-Based Activity Monitors and a Linked Web Portal in an Exercise Referral Scheme: Feasibility Randomized Controlled Trial. J. Med. Internet Res..

[34] Feeley N., Cossette S., Cote J., Heon M., Stremler R., Martorella G., Purden M. (2009). The importance of piloting an RCT intervention. Can. J. Nurs. Res..

[35] Orsmond G.I., Cohn E.S. (2015). The Distinctive Features of a Feasibility Study: Objectives and Guiding Questions. OTJR.

[36] Close A.G., Dreyzin A., Miller K.D., Seynnaeve B.K.N., Rapkin L.B. (2019). Adolescent and young adult oncology-past, present, and future. CA Cancer. J. Clin..

[37] Kolar A.S., Patterson R.E., White E., Neuhouser M.L., Frank L.L., Standley J., Potter J.D., Kristal A.R. (2005). A Practical Method for Collecting 3-Day Food Records in a Large Cohort. Epidemiology.

[38] Kipnis V., Subar A.F., Midthune D., Freedman L.S., Ballard-Barbash R., Troiano R.P., Bingham S., Schoeller D.A., Schatzkin A., Carroll R.J. (2003). Structure of dietary measurement error: Results of the OPEN biomarker study. Am. J. Epidemiol..

[39] Moshfegh A.J., Rhodes D.G., Baer D.J., Murayi T., Clemens J.C., Rumpler W.V., Paul D.R., Sebastian R.S., Kuczynski K.J., Ingwersen L.A. (2008). The US Department of Agriculture Automated Multiple-Pass Method reduces bias in the collection of energy intakes. Am. J. Clin. Nutr..

[40] Burrows T.L., Martin R.J., Collins C.E. (2010). A systematic review of the validity of dietary assessment methods in children when compared with the method of doubly labeled water. J. Am. Diet Assoc..

[41] Burrows T.L., Ho Y.Y., Rollo M.E., Collins C.E. (2019). Validity of Dietary Assessment Methods When Compared to the Method of Doubly Labeled Water: A Systematic Review in Adults. Front. Endocrinol..

[42] Hughes A.R., Summer S.S., Ollberding N.J., Benken L.A., Kalkwarf H.J. (2017). Comparison of an interviewer-administered with an automated self-administered 24 h (ASA24) dietary recall in adolescents. Publ. Health Nutr..

[43] Kirkpatrick S.I., Guenther P.M., Douglass D., Zimmerman T., Kahle L.L., Atoloye A., Marcinow M., Savoie-Roskos M.R., Dodd K.W., Durward C. (2019). The Provision of Assistance Does Not Substantially Impact the Accuracy of 24-Hour Dietary Recalls Completed Using the Automated Self-Administered 24-H Dietary Assessment Tool among Women with Low Incomes. J. Nutr..

[44] Raffoul A., Hobin E.P., Sacco J.E., Lee K.M., Haines J., Robson P.J., Dodd K.W., Kirkpatrick S.I. (2019). School-Age Children Can Recall Some Foods and Beverages Consumed the Prior Day Using the Automated Self-Administered 24-Hour Dietary Assessment Tool (ASA24) without Assistance. J. Nutr..

[45] Jette A.M. (2006). Toward a Common Language for Function, Disability, and Health. Phys. Ther..

[46] Tanner L., Keppner K., Lesmeister D., Lyons K., Rock K., Sparrow J. (2020). Cancer Rehabilitation in the Pediatric and Adolescent/Young Adult Population. Semin. Oncol. Nurs..

[47] Klika R., Tamburini A., Galanti G., Mascherini G., Stefani L. (2018). The Role of Exercise in Pediatric and Adolescent Cancers: A Review of Assessments and Suggestions for Clinical Implementation. J. Funct. Morphol. Kinesiol..

[48] Academy of Pediatric Physical Therapy List of Pediatric Assessment Tools Categorized by ICF Model. https://pediatricapta.org/includes/fact-sheets/pdfs/13%20Assessment&screening%20tools.pdf?v=1.1.

[49] Centers for Disease Control and Prevention Body Mass Index (BMI). https://www.cdc.gov/healthyweight/assessing/bmi/index.html.

[50] Yurkiewicz I.R., Simon P., Liedtke M., Dahl G., Dunn T. (2018). Effect of Fitbit and iPad Wearable Technology in Health-Related Quality of Life in Adolescent and Young Adult Cancer Patients. J. Adolesc. Young Adult Oncol..

